# A Review of the Phytochemistry and Biological Activities of *Echinopsis Radix*

**DOI:** 10.3390/molecules29102267

**Published:** 2024-05-11

**Authors:** Na Sun, Yujing Wang, Shuo Ma, Huanhuan Kang, Caihong Zhou, Linxuan Jin, Xin Zhang, Yu Zhang, Yuhan Yuan, Penghua Shu

**Affiliations:** Key Food and Pharmacy College, Xuchang University, Xuchang 461000, China

**Keywords:** *Echinopsis Radix*, *Echinops latifolius* Tausch, *Echinops grijsii* Hance, Chemical constituents, Biological activity

## Abstract

The main varieties of *Echinopsis Radix* recorded in the Chinese Pharmacopoeia are the roots of *Echinops latifolius* Tausch or *Echinops grijsii* Hance. However, the chemical constituents and biological activities of this herb have not been reviewed. In order to clarify the chemical constituents of the main varieties of this herb and improve the quality of Chinese medicinal material resources, this paper systematically reviewed their chemical constituents and related biological activities. Phytochemical investigations reveal eighty-five compounds including fort y-nine thiophenes (**1**–**49**), eight flavonoids (**50**–**57**), seven caffeic acids and its derivatives (**58**–**64**), eight sesquiterpenoids (**65**–**72**), and thirteen triterpenoids and other compounds (**73**–**85**) were reported from *Echinopsis Radix*. The review of biological activities suggests that thiophenes are the main secondary metabolites of the medicinal material which exert antitumor, insecticidal and antifungal activities. In addition, caffeic acid and its derivatives and sesquiterpenes are potential active ingredients worthy of further study. This review provides an important scientific basis for the development of active ingredients and resource quality evaluation of *Echinopsis Radix*.

## 1. Introduction

As is known to all, the Asteraceae family is one of the biggest families in the world. And the related reports have revealed that it comprises 1000 genera and 25,000–30,000 species, which are widely distributed around the world [1]. Among them, there are over 200 genera and 2000 species in China, and widely distributed throughout the country. It is worth noting that the Asteraceae plant “Loulu”, as a traditional Chinese medicine, has a long history of application in classic prescriptions. As reported, it is commonly used in clinical practice for breast abscess swelling and pain, carbuncle and back pain, scrofula toxin, milk obstruction, and dampness and numbness [2,3]. However, for many years, there has been controversy over the textual research of “Loulu”, especially “Qizhou Loulu” and “Yuzhou Loulu”. Modern researches have shown that “Qizhou Loulu” and “Yuzhou Loulu” are plants of the same family but different genera, and their active ingredients and pharmacological effects are different. Therefore, the two mentioned plants above are recorded separately in the 2020 edition of the Chinese Pharmacopoeia, namely, “Loulu” refers to the dry roots of *Rhaponticum uniflorum* (L.) DC., which is the genus *Rhaponticum*, and “Yuzhou Loulu” also named *Echinopsis Radix* refers to the dry roots of *Echinops latifolius* Tausch or *Echinops grijsii* Hance, which is the genus *Echinops* [4,5,6]. As is well known, the chemical composition and biological activity of traditional Chinese medicine are important indicators for identifying and evaluating the quality of medicinal materials. However, there is currently no systematic review on the chemical composition and biological activity of *Echinopsis Radix* (“Yuzhou Loulu”).

As a famous folk medicine, *Echinopsis Radix* is a perennial herbaceous plant. And it is widely distributed in Gansu, Hebei, Henan, and Liaoning provinces of China. It can exert functions such as clearing heat and detoxifying, expelling pus and stopping bleeding, and eliminating carbuncle and lower breast. It is mainly used for the treatment of ulcers, abscesses, skin heat toxicity, and milk obstruction. According to the description of *Echinopsis Radix* (“Yuzhou Loulu”) in the 2020 edition of the Chinese Pharmacopoeia, the plants also include *Echinops latifolius* Tausch and *Echinops grijsii* Hance [7,8]. Furthermore, it is worth mentioning that in the Flora of China, the Latin name for “*Echinops latifolius* Tausch” has now been revised to *Echinops davuricus* Fisch. ex Hornem [9]. In recent years, there have been increasing reports on the chemical composition and biological activity of *Echinopsis Radix*, but there is no review report on this medicinal herb. In summary, this paper provides a review of the chemical components and biological activities reported in the key varieties of *Echinopsis Radix*, with the aim of providing reference for resource quality evaluation and further development of novel natural lead compounds in *Echinopsis Radix*.

## 2. Methodology

**Figure 1:**
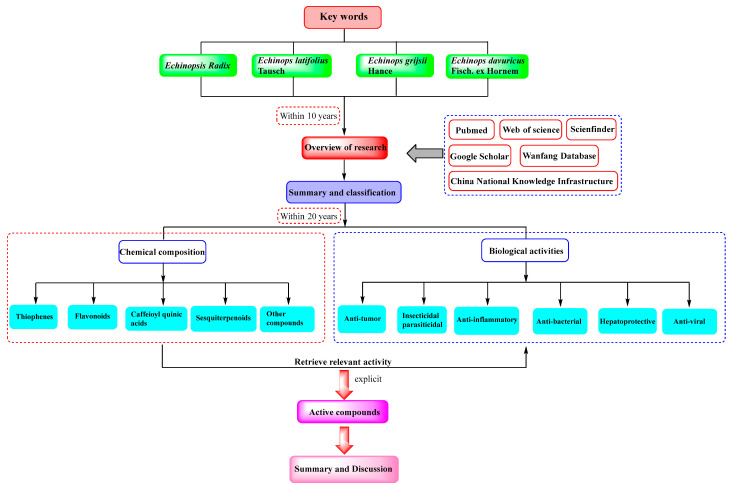
Literature retrieval process.

The relevant studies were searched through PubMed, Web of science, Scienfinder, Google Scholar, Wanfang, and China National Knowledge Infrastructure databases. Firstly, *Echinopsis Radix* (“Yuzhou Loulu”), chemical composition, and biological activity were used as key words for retrieval, and the search time was set to within 10 years. The results showed that there were no comprehensive reports on the chemical composition and biological activity of *Echinopsis Radix* (“Yuzhou Loulu”). Subsequently, papers from the past 20 years have been indexed with *Echinopsis Radix* (“Yuzhou Loulu”), *Echinops latifolius* Tausch, *Echinops grijsii* Hance, and *Echinops davuricus* Fisch. ex Hornem as key words. Moreover, chemical composition and biological activity were further screened as keywords. Then, the chemical composition is classified and summarized according to types thiophenes, flavonoids, caffeoyl quinic acids, sesquiterpenoids, and other compounds, including compound names and structural diagrams. And, the corresponding biological activities of compounds have also been searched and summarized, including anti-tumor, insecticidal, anti-inflammatory, anti-bacterial, hepatoprotective activity and AKR1B10 inhibitory, as well as anti-viral activity. Finally, we systematically reviewed the chemical composition of *Echinopsis Radix* included in the Chinese Pharmacopoeia and discussed the corresponding biological activities of the monomer compounds. The literature retrieval process is as shown in the following Figure 1.

## 3. Phytochemicals

So far, thiophene sesquiterpenes, flavonoids, caffeic acids and their derivatives have been reported from *Echinopsis Radix*. Specially, various types of thiophene have been reported like monothiophene, dithiophene, triphenylthiophene, and tetrathiophene. As presented in Table 1, eighty-five compounds were isolated and identified through various chromatographic and spectroscopic techniques. It is widely known that thiophene compounds are typical secondary metabolites of *Echinopsis Radix*. The root of the plant is the main source of the thiophenes, while most of the sesquiterpenes, caffeic acid, and flavonoids have also been reported from the aerial part of the plant.

### 3.1. Thiophenes

Thiophenes are the most common chemical constituents in the genus *Echinops*. So far, a total of forty-nine thiophenes have been reported from *Echinopsis Radix*. They are classified as eleven monothiophenes (**1**–**11**), twenty-seven dithiophenes (**12**–**38**), six trithiophenes (**39**–**44**), and five dithiophene dimers (**45**–**49**).

**Figure 2:**
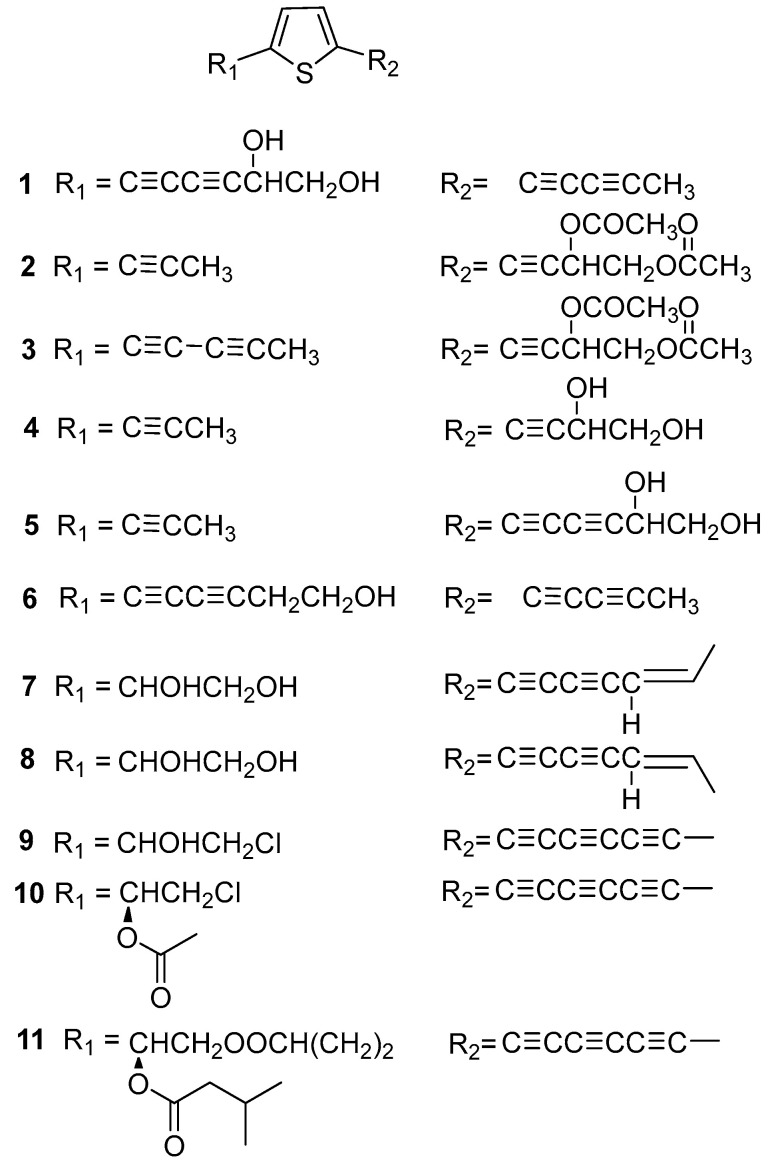
Monothiophenes reported from *Echinopsis Radix*.

Eleven monothiophenes were isolated from *Echinopsis Radix*. As shown in Figure 2, the C-2 and C-5 positions of these compounds are usually substituted by fatty hydrocarbon or ester groups. It is worth noting that most aliphatic hydrocarbon substituents typically contain alkyne functional groups. Among them, compounds **7** and **8** are a pair of cis trans isomers of olefins isolated from the roots of *E. latifolius* [10]. In addition, compounds **9**–**11** were three polyacetylene monothiophenes isolated from the roots of *E. grijsii* [11]. And compounds **9** and **10** are two chlorinated halogenated monothiophenes.

**Figure 3:**
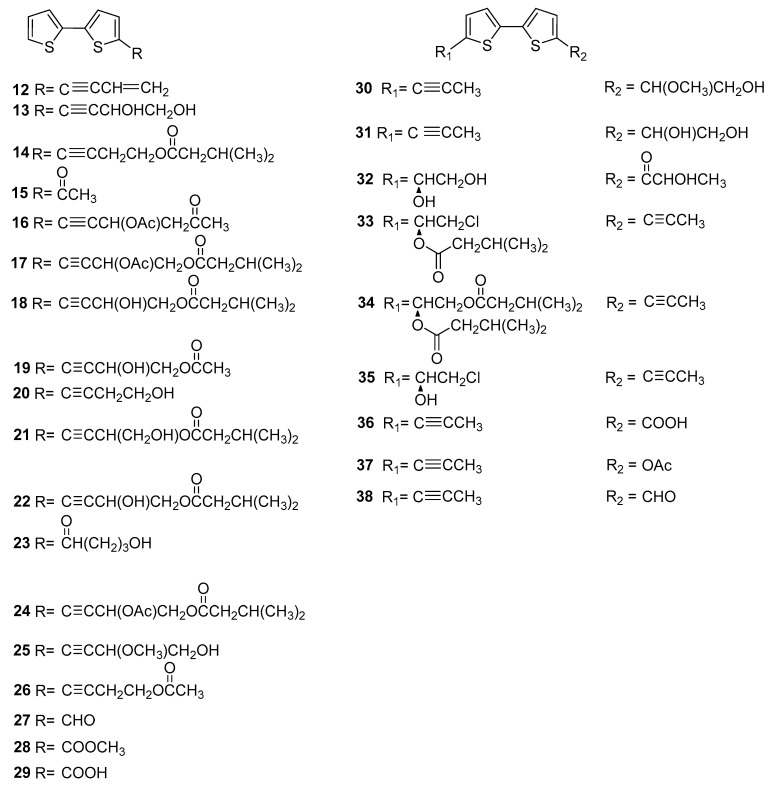
Dithiophenes reported from *Echinopsis Radix*.

As we all know, thiophenes are biosynthetically derived from fatty acids and reduced sulphur. Most of the thiophenes comprised two thiophene rings in their structure and connected through C-2 and C-2′, namely bithiophene. As presented in Figure 3, a total twenty-seven dithiophenes were reported from *Echinopsis Radix*. Among them, they are eighteen mono substituted with C-5 position (**12**–**29**) and nine double substituted with C-5 and C-5′ positions (**30**–**38**). Most of these compounds are lipophilic, with ester, acyl, and hydroxyl groups mainly present in the side chains. Compounds **15**, **21**, and **24** were firstly isolated from the roots of *E. latifolius* by Wang Yi’s research team [12,13]. And compound **25** with the 4-hydroxy-3-methoxy-1-butyny side chain was firstly isolated from the roots of *E. grijsii* [14]. Compounds **32**–**34** are three new disubstituted thiophenes reported for the first time from the roots of *E. grijsii* [11]. In addition, compounds **33** and **35** are two chlorine containing disubstituted thiophenes.

**Figure 4:**
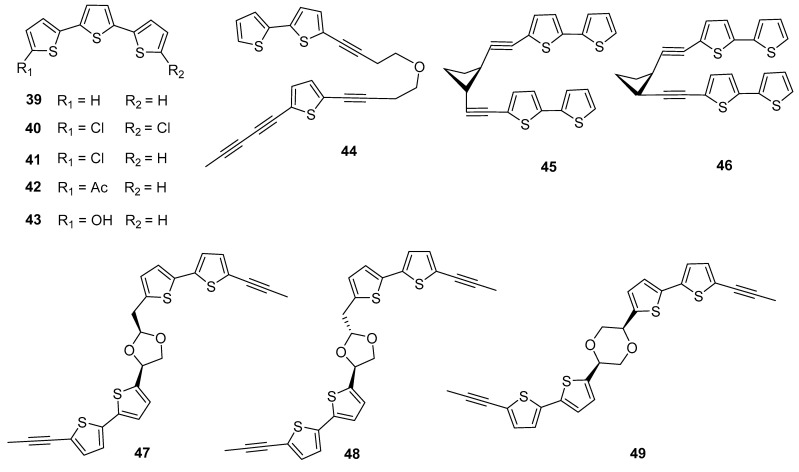
Tricyclic and tetracyclic thiophenes reported from *Echinopsis Radix*.

Furthermore, some thiophene compounds with more than two thiophene rings have also been reported from *Echinopsis Radix*. Among them, the triphenylthiophene compound α-terthienyl (**39**) was firstly isolated from *E. grijsii* [15]. Triphenylthiophenes are not commonly found in the genus *Echinops*, and only five have been isolated from *Echinopsis Radix* [16]. Among them (Figure 4), four compounds (**40**–**43**) with C-5 or C-5′ positions substituted by chlorine, acetyl, or hydroxyl groups, and all were isolated from *E. grijsii*. Interestingly, a trithiophene compound containing three thiophene rings (**44**) has been firstly reported from *E. latifolius*, although it is a derivative of the polymerization of monothiophene and bithiophene [17]. Additionally, five dithiophene dimers have been reported (**45**–**49**), which are connected by carbon-carbon bond or carbon-oxygen bond [18,19,20]. Among them, cardopatine (**45**) and isocardopatine (**46**) are a pair of epimers, while echinbithiophenedimers A (**47**) and B (**48**) are a pair of epimers. And compounds **47**–**49** possessed new carbon skeletons are the first examples of bithiophene dimers furnished by different cyclic diethers. Importantly, compounds **47** and **48** feature an unprecedented 1,3-dioxolane ring system and compound **49** features an unusual 1,4-dioxane ring. The structural types of thiophene compounds of *Echinopsis Radix* are enriched via these compounds, which make good examples of novel thiophene polymers in natural products.

### 3.2. Flavonoids

**Figure 5:**
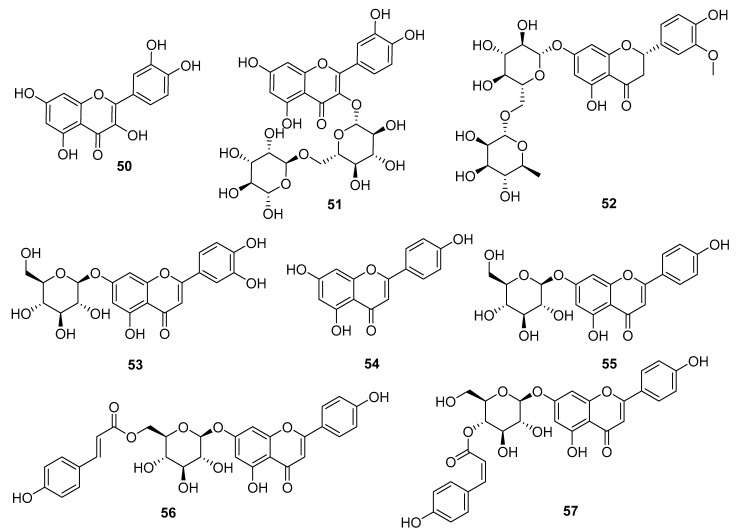
Flavonoids reported from *Echinopsis Radix*.

Although flavonoids are relatively common in most plants, only eight flavonoids (**50**–**57**) have been reported from *Echinopsis Radix* (Figure 5). And all these flavonoids and their glycosides were isolated from the aboveground portion of the plant. For example, compounds **50**–**55** were reported from the aerial part of *E. grijsii* [21], while the glycoside derivatives of flavonoids **56** and **57** were first reported from the flowers of *E. grijsii* [22].

### 3.3. Caffeoyl Quinic Acids

**Figure 6:**
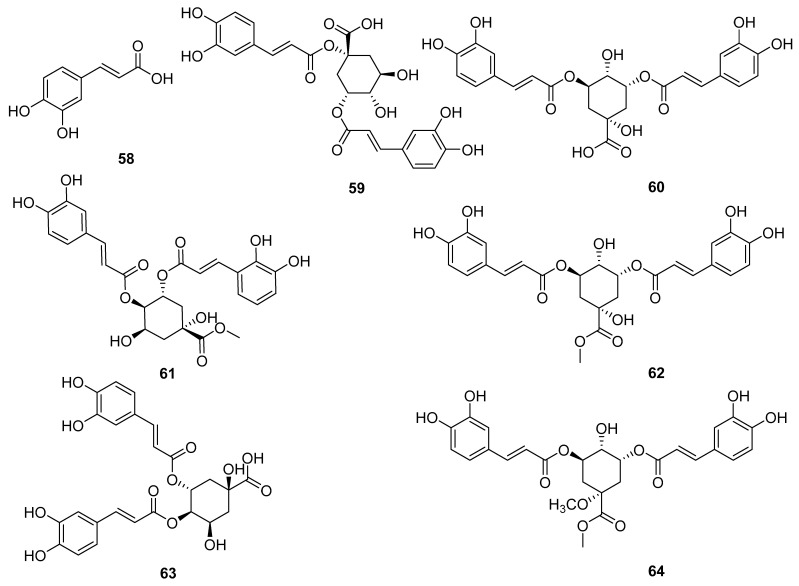
Caffeoyl quinic acids reported from *Echinopsis Radix*.

Caffeic acid and its derivatives are very rare in the entire genus *Echinops*. So far, seven caffeic acid compounds (**58**–**64**) have been obtained by Xue Pei-feng et al. from the flowers of *E. latifolius* [22]. These compounds are not only the first to be obtained from this plant, but also the first to be discovered from the genus *Echinops*. As shown in Figure 6, compounds **59**–**64** are both obtained by esterification of one quinic acid with two caffeic acids.

### 3.4. Sesquiterpenoids

**Figure 7:**
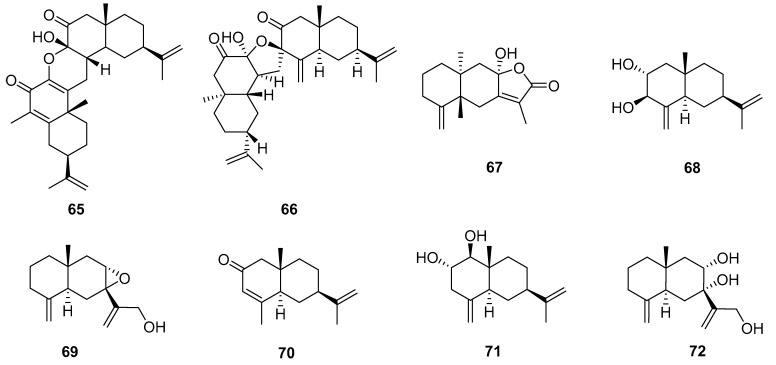
Sesquiterpenoids reported from *Echinopsis Radix*.

Eight sesquiterpenoid compounds were obtained from *Echinopsis Radix* and classified as two sesquiterpene dimers (**65** and **66**) and six eudesmane-type sesquiterpenes (**67**–**72**) (Figure 7). Compounds **65** and **67** were isolated for the first time from the methanol extract of the root of *E. latifolius* [10]. And compound **65** is an unprecedented new carbon skeleton dimer sesquiterpene formed by the cyclization of two eudesmane-type sesquiterpenes. Compounds **66** and **68**–**72** were isolated from the ethyl acetate extraction portion of the root of *E. grijsii* [23]. And compound **66** represented a totally new carbon skeleton of the disesquiterpenoid, featuring a unique 6/6/5/6/6 ring system with an oxaspiro unit. Additionally, the biogenetically possible precursors of **66** suggested that it could be produced by incorporating two analogues of oxygenated eudesmanes. Especially, sesquiterpenes and sesquiterpene dimers of *Echinopsis Radix* are a class of components that deserve attention.

### 3.5. Other Compounds

**Figure 8:**
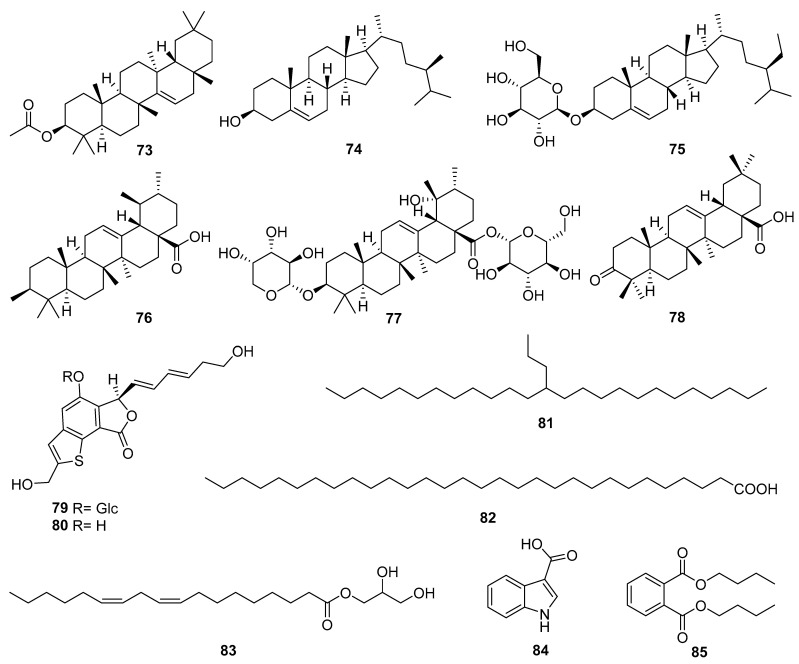
Other compounds reported from *Echinopsis Radix*.

In addition to the chemical components reported above, *Echinopsis Radix* is also rich in many other components, such as triterpenes, steroids, benzothiophenes, and fatty acids [24,25,26]. The structures of other compounds (**73**–**82**) are presented in Figure 8. Compounds **73**, **76**–**78** are four pentacyclic triterpenoid compounds, of which **73** and **78** belong to the oleanolane type, while **76** and **77** belong to the ursulane type. Steroids **74** and **75**, as well as fatty acids **81** and **82** are widely present in various plants and have been reported in the roots or flowers of *E. latifolius*. Compounds **79** and **80** are the only two benzothiophene glycosides and aglycones discovered from the roots of *E. grijsii*. Furthermore, in our previous study on the aboveground part of *E. davuricus*, compounds **83**–**85** were isolated for the first time.

## 4. Biological Activities

As a traditional Chinese medicine, *Echinopsis Radix* exhibits various pharmacological activities, such as anti-tumor, antiviral, anti-HIV, anti-bacterial, insecticidal and parasiticidal effects, liver protection, immune regulation, and anti-inflammatory effects [24]. However, due to its complex chemical composition, most of the reported pharmacological effects are crude extracts or mixtures, and the chemical structure is not yet clear. In recent years, with the continuous deepening of research on the chemical composition of *Echinopsis Radix*, the biological activity of monomer compounds has also been reported. Therefore, it is necessary to summarize the biological activity of monomeric compounds from *Echinopsis Radix*.

### 4.1. Anti-Tumor Activity

Early studies have shown that the crude extract of *Echinopsis Radix* exhibits excellent anti-tumor activity. In recent years, the main anti-tumor active ingredients have also been reported. Wang et al. reported the important cytotoxic activities of compounds **23** and **45** on human melanoma cells (A375-S2) and cervical cancer cells (Hela), with IC_50_ values from 3.1 to 13.5 µmol/L. And the phototoxicity of two thiophene compounds is lower than that of α-terthienyl with stronger activity [17]. The study results of Zhang et al. revealed that compounds **1**, **5**, **6**, **12**, **19**, **31**, and **80** exhibit good cytotoxic activity against leukemia cell lines HL60 and K562, with IC_50_ values from 0.23 to 30.6 µg/mL. Among them, the cytotoxicity of monothiophenes **1** (with IC_50_ values 0.23 and 0.47 µg/mL) and **6** (with IC_50_ values 0.27 and 0.45 µg/mL) are much higher than that of other thiophenes, [24]. And the substituted alkyne groups on both sides of a single thiophene may be a key factor in enhancing cytotoxic activity. Jin et al. reported the cytotoxicity of compounds **2**, **3**, **12**, **14**, **16**, **17**, and **39** on four types of tumor cells (hepatocellular carcinoma cells HepG2, leukemia cells K562 and HL60, breast cancer cells MCF-7) based on tracking the active components of dichloromethane. Among them, compounds **2**, **14**, and **16** showed high cell inhibitory rate against HL60 (with IC_50_ values 10, 8 and 12 µg/mL, respectively), compounds **17** and **39** showed major inhibitory activity against K562 (with IC_50_ values 12 and 7 µg/mL), and compounds **16** and **17** are two main inhibitory influences on cytotoxicity against HepG2 (with IC_50_ values 10 and 1.8 µg/mL) [27]. In summary, the cytotoxicity of thiophene is related to thiophene polymerization and side chain substitution, and acyl substituted dithiophenes exhibit stronger anti-tumor activity than α-trithiophene and monothiophene.

### 4.2. Anti-Inflammatory Activity

Although there are many reports on the anti-inflammatory activity of *Echinopsis Radix*, they all focus on the crude extract [28,29]. However, there are relatively few reports on monomeric compounds that exhibit anti-inflammatory activity in the plants. At present, only five anti-inflammatory active compounds have been reported from *Echinopsis Radix*. In 2015, The research results of Chang Fangpin et al. revealed that compounds **1**, **13**, and **20** exhibited significant inhibitory activity against nitrite of LPS-stimulated production in the RAW 264.7 cell line. And the IC_50_ values were 2.5, 20.0 and 6.7 μg/mL, respectively [14]. Subsequently, Jin Qinghao et al. discovered the important anti-inflammatory activities of compounds **7**, **8**, **30**, and **31**, with IC_50_ values of 12.8–48.7 μM. [10]. The structure-activity relationship analysis suggested that the anti-inflammatory activity of monothiophene is stronger than that of dithiophene, while the activity of double substituted dithiophene is better than that of single substituted dithiophene. In addition, the presence of cis double bonds in monothiophene enhances anti-inflammatory activity.

### 4.3. Hepatoprotective Activity

In early pharmacological studies, Lin et al. reported that *Echinopsis Radix* has a significant hepatoprotective effect, which can improve CCl4 induced liver necrosis and dysfunction in rats [30]. Subsequently, Li Xifeng et al. demonstrated that the hepatoprotective effect of the ethanol extract may be related to its ability to scavenge free radicals and resist oxidation in the body [31]. Until 2010, Shi jing et al. first reported that compound **1** possessed potent NAD(P)H: quinone oxidoreductase1 (NQO1) inducing activity and could activate Keap1-Nrf2 pathway effectively in murine hepatoma Hepa 1c1c7 cells [32]. In addition, our previous chemical and biological activity studies revealed that the important anti-cancer targets of *Echinopsis Radix*, especially the anti-liver cancer AKR1B10, by network pharmacology strategies. And for the first time, compounds **83** and **85** have been reported to have significant AKR1B10 inhibitory activity, which provides new ideas for later research on the hepatoprotective effect of *Echinopsis Radix* [26].

### 4.4. Insecticidal and Parasiticidal Activity

Natural thiophene compounds show obvious insecticidal effects, such as phototoxic activity against bacteria, yeasts, insects and various experimental microorganisms. The mosquito-killing effect of α-T (compound **39**) was firstly reported by Nivsarkar et al. [33], and then the insecticidal effects of other thiophenes have also been reported. In 2017, Zhao Meiping et al. obtained three compounds **12**, **14**, and **39** insecticidal activities against mosquito larvae based on activity directed separation. Among them, **12** and **14** possessed sound larvicidal activity against the fourth instar larvae of *Ae. albopictus* with LC_50_ values of 0.34 g/mL and 0.45 g/mL, respectively. And **12** and **14** had LC_50_ values against the fourth instar larvae of *An. sinensis* of 1.36 g/mL and 5.36 g/mL, respectively. In addition, **12** and **14** also possessed LC_50_ values against the fourth instar larvae of *C. pipiens pallens* of 0.12 g/mL and 0.33 g/mL, respectively [34]. In 2019, Liu Tingting et al.’s research revealed that compounds **9**, **10**, **30**, **32**, **33**, **35**, **37**, and **38** showed stronger nematicidal activity against *Meloidogyne incognita* than commercial nematicide abamectin. And **30**, **32**, **33**, and **35** have been proven to be non phototoxic. Specially, compounds **32** and **35** were the most potent thiophenes against *M. incognita* with LC_50_ values 2.57 and 0.91 μg/mL in light, 1.80 and 0.86 μg/mL in dark, respectively [11]. Subsequently, Liu Tingting et al. also reported that the new framework of sesquiterpene dimers **66** exhibited stronger insecticidal activity than sesquiterpene compounds **68**–**72**, and even stronger insecticidal activity against *aphids* than the commercially available aphid fungicide pyrimethadone [23]. Additionally, Wu Haibo et al. demonstrated for the first time that compounds **47**–**49** of dithiophene dimer had better nematicidal activity against *Meloidogyne incognita* than commercial nematicides ethoxyphosphorus, and had no phototoxicity [20]. The above research results indicate that thiophene backbone is essential for nematicidal activity, while disubstituted groups were helpful for nonphototoxicity. And acyl groups can inhibit the effect of light on activity, while chlorine plays an important role in promoting activity. Furthermore, sesquiterpene dimers and dithiophene dimers could be served as novel potential biological insecticides.

### 4.5. Anti-Bacterial Activity

The inhibitory activities of the total extract and each extract of *Echinopsis Radix* against fungi such as *Staphylococcus aureus*, *Escherichia coli*, *Bacillus subtilis*, *Salmonella typhi* and *Pseudomonas aeruginosa* were first reported by Li Xifeng. et al. The results showed that the ethyl acetate and n-butanol extracts were effective parts with antibacterial activity [32]. In the study of plant comprehensive disease complexes, Liu Tingting et al. revealed the excellent antifungal activity of thiophene compounds against six fungi (*F. solani*, *F. oxysporum f.* sp. *vasinfectum*, *F. oxysporum f.* sp. *niveum*, *P. infestans*, *C. gloeosporioides*, and *A. alternate*). Among them, compared with the commercial fungicide carbendazim, compounds **9**, **10**, **31**–**33**, **35**, and **36** exhibited equal or better antifungal activity against six plant pathogenic fungi [23]. Importantly, the antifungal activities of dimeric dithiophene **47**–**49** were first reported by Wu Haibo et al. The results showed that they had good antifungal activity against five plant pathogenic fungi. And compound **49** had antibacterial activity similar to carbendazim against *Fusarium oxysporum* and rice blast fungus, with a minimum inhibitory concentration of 8 μg/mL [20]. The structure-activity relationship analysis suggests that thiophene substituted with chlorine has the strongest antibacterial activity, and dimeric dithiophene is an important potential antifungal agent.

### 4.6. Anti-Viral Activity

So far, there are few reports on the antiviral activity of *Echinopsis Radix*. Only triple-thiophene compound **39** has been shown to have significant anti-HIV activity. Although the activity of a decreased in the presence of bovine serum, it could inactivate 104 or 105 viral vectors at a mass concentration of 0.1 mg/mL [35]. Therefore, the antiviral activities of triple-thiophene compounds and their derivatives are worthy of further study.

## 5. Conclusions

*Echinopsis Radix* (“Yuzhou Loulu”) as well as *E. latifolius*, *E. grijsii*, and *E. davuricu* is are important resource of traditional Chinese medicine. This review not only shows their rich chemical composition and diverse biological activities, but also demonstrates their common chemical composition and similar biological activities. And thiophene compounds with various types including monothiophene, dithiophene, trithiophene, and dimeric dithiophene, are typical secondary metabolites. Biological activity survey reveals that the most abundant bioactive secondary metabolites in this medicinal herb are thiophene compounds, which are mentioned as responsible for the anti-tumor, insecticidal, and anti-fungal effects observed. Significantly, the discussion of structure-activity relationships lays the foundation for the development of active compounds. In addition, it has been observed that the potential uses of *Echinopsis Radix* in the hepatoprotective effect and antiviral activity have not been scientifically addressed yet. This review not only clarifies the main chemical components of *Echinopsis Radix* for the quality evaluation, but also provides scientific ideas for further exploration of its active ingredients.

## Figures and Tables

**Table 1:** The names of compounds **1**–**85** isolated from *Echinopsis Radix*.

No.	Name	No.	Name
**1**	2-(3,4-dihydroxybut-1-ynyl)-5-(penta-1,3-diynyl)-thiophene	**44**	5-{4-[4-(5-pent-1,3-diynylthiophen-2-yl)-but-3-ynyloxy]-but-1-ynyl}-2,2′-bithiophene
**2**	2-(3,4-diacetoxybut-1-ynyl)-5-(propary-1-ynyl)-thiophene	**45**	Cardopatine
**3**	2-(3,4-diacetoxy-but-1-ynyl)-5-(penta-1,3-diynyl)-thiophene	**46**	Isocardopatine
**4**	2-(3,4-dihydroxybut-1-ynyl)-5-(propary-1-ynyl)-thiophene	**47**	echinbithiophenedimer A
**5**	echinoynethiophene A	**48**	echinbithiophenedimer B
**6**	2-(3,4-diacetoxybut-1-ynyl)-5-(propary-1-ynyl)-thiophene	**49**	echinbithiophenedimer C
**7**	5-(1,2-dihydroxy-ethyl)-2-(*E*)-hept-5-ene-1,3-diynylthiophene	**50**	Quercetin
**8**	5-(1,2-dihydroxy-ethyl)-2-(*Z*)-hept-5-ene-1,3-diynylthiophene	**51**	Rutin
**9**	echinothiophene A	**52**	Hesperidin
**10**	echinothiophene B	**53**	luteolin 7-*O*-β-d-glucoside
**11**	echinothiophene C	**54**	Apigenin
**12**	5-(but-3-en-1-ynyl)-2,2′-bithiophene	**55**	apigenin 7-*O*-β-d-(6′′-*P*-coumaroyl)-glucoside
**13**	5-(3,4-dihydroxybut-1-ynyl)-2,2′-bithiophene	**56**	apigenin 7-*O*-β-d-glucoside
**14**	5-(4-isovaleroyloxybut-1-ynyl)-2,2′-bithiophene	**57**	apigenin 7-*O*-β-d-(4′′-P-coumaroyl)-glucoside
**15**	5-acety-2,2′-bithiophene	**58**	caffeic acid
**16**	5-(3,4-diacetoxybut-ynyl)-2,2′-bithiophene	**59**	1,5-*O*-dicaffeoyl quinic acid
**17**	5-(3-acetoxy-4-isopentyloxybut-1-ynyl)-2,2′-bithiophene	**60**	3,5-*O*-dicaffeoyl quinic acid
**18**	5-(3-hydroxy-4-isovaleroyloxybut-1-ynyl)-2,2′-bithiophene	**61**	methyl 3,4-*O*-dicaffeoyl quinic acid
**19**	5-(3-hydroxy-4-acetoxybut-1-ynyl)-2,2′-bithiophene	**62**	methyl 3,5-*O*-dicaffeoyl quinic acid
**20**	5-(4-hydroxy-1-butynyl)-2,2′-bithiophene	**63**	3,4-*O*-dicaffeoyl quinic acid
**21**	5-(3-hydroxy-3-isovaleroyloxybut-1-ynyl)-2,2′-bithiophene	**64**	methyl-1-*O*-methyl 3,5-*O*-dicaffeoyl quinic acid
**22**	5-(3-hydroxy-4-isovaleroyloxybut-1-ynyl)-2,2′-bithiophene	**65**	latifolanone A
**23**	5-(4-hydroxybut-1-one)-2,2′-bithiophene	**66**	atractylenolide-III;
**24**	5-(3-acetoxy-4-isovaleroyloxybut-1-ynyl)-2,2′-bithiophene	**67**	echingridimer A
**25**	5-(4-hydroxy-3-methoxy-1-butyny)-2,2′-bithiophene	**68**	echingriol A
**26**	5-(4-acetoxy-1-butynl)-2,2′-bithiophene	**69**	echingriol B
**27**	5-formyl-2,2′-bithiophene	**70**	eudesmane K
**28**	methyl-2,2′-bithiophene-5-carboxylate	**71**	nardoeudesmol A
**29**	2,2′-bithiophene-5-carboxylicacid	**72**	Rhaponticol
**30**	6-methoxy-arctinol-b	**73**	taraxeryl acetate
**31**	artinol-b	**74**	β-sitosterol
**32**	echinothiophene D	**75**	daucosterol
**33**	echinothiophene E	**76**	ursolic acid
**34**	echinothiophene F	**77**	sanguisorbin-I;
**35**	2-prop-1-inyl-5′-(2-hydroxy-3-chloropropyl)-2,2′-bithiophene	**78**	3-oxooleanolic acid
**36**	artinol	**79**	Echinothiophene
**37**	artinone-b	**80**	Echinothiophenegenol
**38**	artinol	**81**	13-propyl-pentacosane
**39**	α-terthienyl	**82**	triacontanoic acid
**40**	5,5′-dichloro-α-terthienyl	**83**	1-monolinolein
**41**	5-chloro-α-terthienyl	**84**	indole-3-carboxylic acid
**42**	5-acetyl-α-terthienyl	**85**	bis(CE)-hex-2-enyl] carbonate
**43**	5-hydroxy-α-terthienyl		
